# The Potential Distribution of *Pythium insidiosum* in the Chincoteague National Wildlife Refuge, Virginia

**DOI:** 10.3389/fvets.2021.640339

**Published:** 2021-02-19

**Authors:** Manuel Jara, Kevin Holcomb, Xuechun Wang, Erica M. Goss, Gustavo Machado

**Affiliations:** ^1^Department of Population Health and Pathobiology, College of Veterinary Medicine, North Carolina State University, Raleigh, NC, United States; ^2^U.S. Fish & Wildlife Service, Chincoteague National Wildlife Refuge, Chincoteague, VA, United States; ^3^Plant Pathology Department and Emerging Pathogens Institute, University of Florida, Gainesville, FL, United States

**Keywords:** disease mapping, spillover, equine pythiosis, spatial epidemiology, ecological niche model

## Abstract

*Pythium insidiosum* is a widespread pathogen that causes pythiosis in mammals. Recent increase in cases reported in North America indicates a need to better understand the distribution and persistence of the pathogen in the environment. In this study, we reconstructed the distribution of *P. insidiosum* in the Chincoteague National Wildlife Refuge, located on Assateague Island, Virginia, and based on 136 environmental water samples collected between June and September of 2019. The Refuge hosts two grazing areas for horses, also known as the Chincoteague Ponies. In the past 3 years, 12 horses have succumbed to infection by *P. insidiosum*. Using an ecological niche model framework, we estimated and mapped suitable areas for *P. insidiosum* throughout the Refuge. We found *P. insidiosum* throughout much of the study area. Our results showed significant monthly variation in the predicted suitability, where the most influential environmental predictors were land-surface water and temperature. We found that June, July, and August were the months with the highest suitability for *P. insidiosum* across the Refuge, while December through March were less favorable months. Likewise, significant differences in suitability were observed between the two grazing areas. The suitability map provided here could also be used to make management decisions, such as monitoring horses for lesions during high risk months.

## Introduction

*Pythium insidiosum* is the only etiologic agent of pythiosis that affects mainly mammals in tropical and subtropical countries (1, 2). *P. insidiosum* is an oomycete, a eukaryotic lineage in the stramenopiles, and its closest relatives are plant pathogens (3). Pythiosis is characterized by chronic lesions on the cutaneous and subcutaneous, intestinal, and bone tissues, as well as invasion of blood vessels in infected animals and rarely humans (4, 5), which lead to death or render the affected individual lame if left untreated (2, 4).

Pythiosis infection is acquired by animals and plants through the direct contact of wounds with water that contains motile *P. insidiosum* spores (zoospores) (6–10). Zoospores are typically released by sporangia, which are not highly differentiated from hyphae in *P. insidiosum* (2, 4). Previous studies of other oomycetes have demonstrated that zoospores may be specifically attracted to molecules produced by hosts through chemotaxis (11, 12). Zoospores encyst in response to environmental cues, which may include a host substrate, other substrates, or chemical cues (4). The encysted spore produces a germination tube which also uses chemotaxis to find the host (13, 14). Zoosporulation is triggered by environmental and host cues. *Pythium* species vary in their response to cues and some are active in cold temperatures, whereas others, including *P. insidiosum*, can thrive at warm temperatures like 37°C (15).

The ecological requirements of *P. insidiosum* resemble the typical plant pathogens of the genus *Pythium* sensu lato, which tend to be ubiquitous with broad host ranges (4). *P. insidiosum* is found in freshwater bodies with lilies and aquatic grasses as the primary vegetation coverage (4), as well as ephemeral ponds and flooded areas (10, 16). As clinical and public awareness of pythiosis has increased, the number of reported cases in areas previously thought to be inhospitable for *P. insidiosum* is also on the rise (17, 18). In the United States, *P. insidiosum* has been described to be widely distributed, with case reports mostly in dogs and horses (19–22) and rarely in humans (23, 24).

Few studies have examined the spatial distribution of *P. insidiosum* using environmental samples. In Thailand, the pathogen was found in rice fields (10) and in the soil surrounding bodies of freshwater (10, 25). Another study that utilized environmental samples found multiple genetic lineages of *P. insidiosum* in lakes and ponds of north-central Florida (22). A study conducted in the Brazil-Uruguay border regions reconstructed the ecological conditions of *P. insidiosum* circulation on tested positive horses, identifying areas with a potential risk of pythiosis infection (26).

In this study, we utilized environmental water samples and fine-scale geographic and ecological factors to characterize areas suitable for *P. insidiosum* in the Chincoteague National Wildlife Refuge and map its potential distribution. Ultimately, this study aims to provide data for the design of strategies to reduce the risk of exposure of the Chincoteague ponies to *P. insidiosum* within the Refuge.

## Materials and Methods

**Figure 1:**
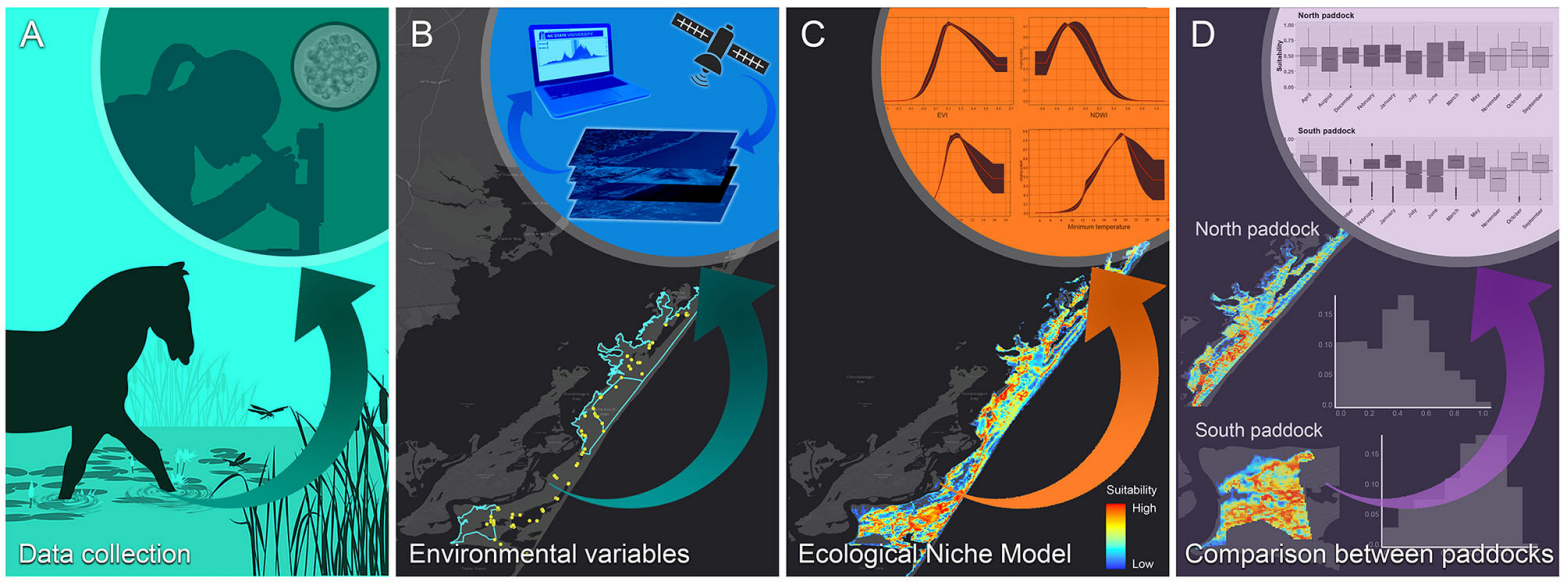
The study workflow. **(A)** Fieldwork for water sample collection and *Pythium insidiosum* laboratory identification; **(B)** gathering of environmental and landscape variables relevant for the Chincoteague National Wildlife Refuge; **(C)** ecological niche model calibration, and **(D)** suitability of the northern and southern grazing areas “paddock” comparison.

The modeling framework used in this study included, (i) data collection, considering the definition of sampling sites for water sample collection, isolation, and identification of *P. insidiosum* (Figure 1A), (ii) extraction of the most suitable environmental variables used in the ecological niche model (ENM) (Figure 1B), (iii) ENM model calibration and evaluation (Figure 1C), followed by the (iv) comparison in the suitable areas predicted by the annual and monthly ENMs in the northern and southern grazing paddocks (Figure 1D).

### Data Collection

The water samples were collected from moist soil management units (also called impoundments), localized ephemeral ponds and watering holes, and tidal salt marsh located within both grazing paddocks throughout the Virginia portion of Assateague Island (Supplementary Figure 1). Sampling was conducted monthly from June, July, and September 2019. Sites with at least one positive result in the laboratory procedures described below were used as presence occurrences (using their geographic coordinates) in the Ecological Niche Model (ENM).

### Samples Processing

**Figure 2:**
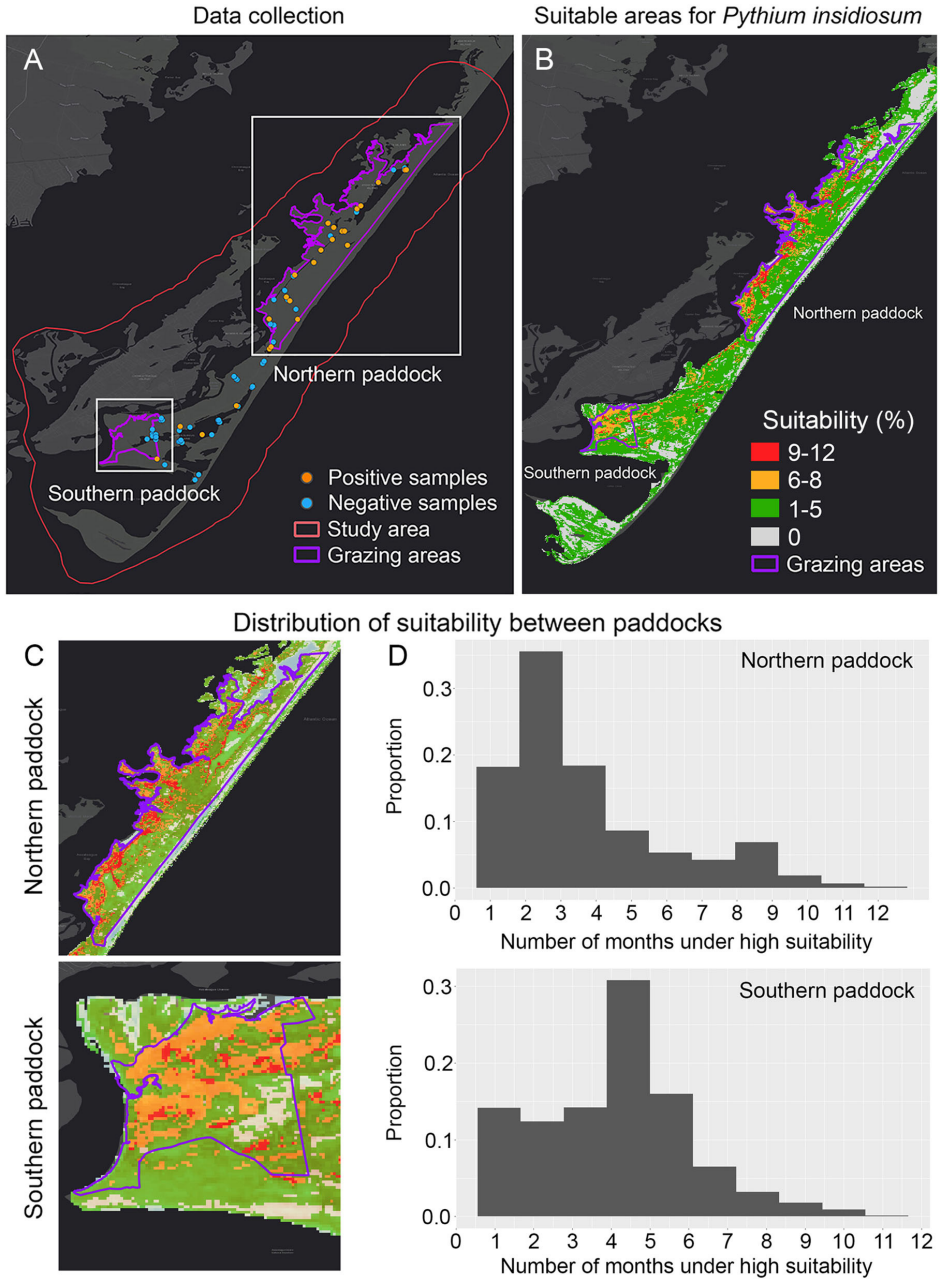
Dynamic ecological niche model showing the most suitable areas of *Pythium insidiosum* in Chincoteague National Wildlife Refuge. **(A)** Shows positive and negative *P. insidiosum* detections in both grazing paddocks, delimited by purple lines. **(B)** Shows the dynamic ENM results and the expanded visualization of each grazing paddock **(C)**. **(D)** Shows the comparison of the predicted suitability weighted distribution for *P. insidiosum* for each paddock, where suitability values range from 0 to 1, with 1 being the most suitable.

At each sampling site (Figure 2A), 500 ml high-density polyethylene (HDPE) bottles with polypropylene screw caps were used to sample water by placing the opening one to three inches below the surface of the water. In addition, water temperature and salinity were measured at each site, using a handheld refractometer for salinity. Immediately or shortly after the collection of water, autoclaved human and dog hairs were added to each collection bottle and stored at room temperature for 1–3 days. On June 24, 34 sites were sampled and hairs were incubated in sampled water for 72 h. Water was removed and bottles were shipped to the University of Florida, where hairs were removed from bottles, blotted on paper towels, and plated on both Sabouraud dextrose agar (Difco) media amended 25 mg/ml pimaricin, 200 mg/ml ampicillin, 10 mg/ml rifampicin, 50 mg/ml PCNB, and 200 mg/ml streptomycin sulfate (SDA-PARPS) and incubated at 37°C. Plates were checked for colonies each day for 3 days. Characteristic Pythium colonies were isolated by serial transfer of the growing edge of the Pythium colony. On July 22–24, two or four 500 ml bottles of water were sampled from each of 59 sites. Hair was incubated in water samples for 24 h at room temperature and immediately transferred to SDA-PARPS. Hairs were dried and plated on SDA media immediately after incubation. Plates were incubated at room temperature for 1–2 days and subsequently at 37°C, and checked for colonies each day. On September 20, 36 sites were sampled using two water bottles per site. Hair was incubated for 72 h at room temperature, after which isolation of *Pythium* proceeded as in July.

On SDA media, *P. insidiosum* exhibits a distinct colony morphology compared to other common *Pythium* species. PCR identification was also used for all isolated *Pythium* colonies. A small amount of mycelia was collected from 2 day-old *Pythium* cultures and genomic DNA extracted from the mycelia using the REDExtract-N-Amp kit (Sigma-Aldrich): mycelia were placed in 50 μl extraction solution in a 0.2 ml sterile PCR tube, vortexed well, and then incubated at 95°C for 10 min. After incubation, 50 μl dilution solution was added and vortexed again. Extracted genomic DNA was stored at 4°C. To confirm successful DNA extraction, the ITS region was first amplified using ITS1 and ITS4 primers (27). The PCR reaction used 4.4 μl water, 10 μl Red Extract-N-Amp PCR ReadyMix (Sigma-Aldrich), 0.8 μl each of each primer at 10 μm, and 4 μl extraction products. The PCR conditions were initial denaturation at 94°C for 3 min, 35 cycles of denaturation at 94°C for 1 min, annealing at 55°C for 1 min, and extension at 72°C for 1 min followed by a final extension at 72 °C for 10 min. Bands were confirmed on a 1% agarose gel. Once successful amplification of DNA was confirmed, the extraction products were used for a second PCR with primers ITSpy1 and ITSpy2 to positively identify *P. insidiosum* (10). The PCR reaction used 2 μl water, 10 μl Red Extract-N-Amp PCR ReadyMix, 2 μl of each primer at 2 μm, and 4 μl extraction products. The PCR conditions were initial denaturation at 94°C for 3 min, 35 cycles denaturation at 94°C for 45 s, annealing at 60°C for 30 s, and extension at 72°C for 30 s, followed by a final extension at 72°C for 10 min. The ITSpy PCR products were run on a 1.5% agarose gel. Each ITSpy PCR was conducted with positive control *P. insidiosum* isolate NATL_5A, collected in Gainesville, Florida (22). Selected ITS PCR products were sequenced to confirm the ITSpy PCR-based identification. Single-stranded DNAs were removed using 0.5 μl ExoSAP-IT (Affymetrix) and sent to Genewiz, LLC (South Plainfield, NJ) for Sanger sequencing.

### Ecological Niche Model (ENM)

The variables used to estimate the distribution of *P. insidiosum* were selected based on previous studies that have described the environmental requirements of this oomycete (22, 26, 28), including the pathogen preferences for survival in specific landscapes with high humidity, and high temperature (15, 29, 30). To reconstruct the environment settings where *P. insidiosum* is more likely to be distributed across the island (31, 32), we used land-surface water, soil cation exchange capacity (a proxy of soil salinity), mean temperature, minimum temperature, and maximum temperature. These variables were collected and processed at 30 m scale, which is considered a fine-scale in remote sensing. Temperature variables (mean, minimum, and maximum) correspond to land surface temperature data. To assess land-superficial water bodies, we used normalized-difference water index (NDWI) (33) because it has been successfully used in several surface-water body classification methods (34–37). Soil salinity was estimated using soil cation exchange capacity as a proxy (38, 39). For all variables, but soil salinity, we reconstructed monthly averages from June 2019 until May 2020 (Supplementary Table 1). Soil salinity was available only at annual average. We used VIF (Variance Inflation Factors) to identify and exclude correlated variables before ENM models were built. VIF > 7 was considered as exclusion criteria. VIF was calculated using the “usdm” package (40) see, Supplementary Table 1 for more details.

ENMs were conducted using MaxEnt v3.4.1 (41), a maximum entropy-based machine learning software that uses occurrence-background algorithms to estimate the probability spatial distribution for a species' occurrence across a selected calibration region, based on environmental constraints and assumptions (42, 43). MaxEnt models were performed with clamping and extrapolation turned off (i.e., no prediction outside the range of environmental conditions in the calibration data) (44–46). To determine the model parametrization with the best fit to the data available, we assessed MaxEnt models under a range of regularization multiplier values (0.1, 0.3, 0.5, 0.7, 0.9, 1.3, 1.5, 1.7, 1.9, and 2) (47). At the same time, we explored all the possible feature combinations available in MaxEnt, from a single feature, linear (L), quadratic (Q), product (P), threshold (T), and hinge (H) using the “ENMeval” package (48). Models were selected based on Akaike Information Criterion (AIC) (49), where lower values represent a better model fit to the data with moderate model complexity. We chose as the final model the one with ΔAICc = 0 from among the 319 model combinations generated in ENMeval (48) for each feature and regularization used (47) (Supplementary Table 2). To facilitate interpretations of final models, we used the MaxEnt logistic output as a proxy of environmental suitability (43). Briefly, the logistic output of the whole study area was cropped by the two defined grazing areas: the northern and southern grazing areas (paddocks). The northern paddock is 13,767 km^2^ and holds ~100 horses, and the southern paddock holds ~40 horses in an area of 2,215 km^2^. To extract the “suitability values,” we used the sample raster values tool in QGIS v3.10.5. We extracted the suitability value for each month and for the whole year within each paddock and compared their weighted distribution. The suitability distribution was simplified by dividing it by the number of cells (30 m resolution) of each paddock: 18,797 cells in the north, and 3,086 in the south paddock. To assess the difference between suitability distributions between the paddocks we used the Two-sample Welch T-statistic (50) using the “dplyr” package (51) in R software v3.5.1 (52).

The ENM final model was calibrated in July and then projected to the remaining months using the remote sensing monthly variables mentioned above, as well as the features and regularization parameters resulting from the model calibration. These ENMs projections were conducted via model transference considering MaxEnt model with extrapolation and clamping turned off (46). To determine how each environmental variable affects the ENM predictions, the response curves of the annual model was recovered (Supplementary Figure 2). The monthly ENMs were transformed into binary predictions using a threshold removing 10% of the calibration occurrences (error = 10%) to facilitate the interpretation of the results as well as reducing the uncertainty in the estimations (53). The resulting monthly binary models were assembled by adding the binary predictions to generate a final continuous model ranging from low (1) to high (12) values.

### Characterization of the Most Suitable Areas

Using the binary maps mentioned above, we extracted the land use information to identify the landscape conditions that are related to the most suitable areas for *P. insidiosum*. The land cover information used here comes from the Multi-Resolution Land Characteristics (MRLC) consortium (54).

## Results

In June, nine of 40 sites produced *P. insidiosum* positive samples, confirmed by ITSpy PCR. In July, 16 of 50 sites produced *P. insidiosum* colonies, confirmed by ITSpy PCR. Of the 16 sites, 9 were from sites where four 500 ml bottles of water were collected and 7 were from sites where two bottles were collected. Only three sites of 36 produced *P. insidiosum* colonies in September, two confirmed by ITSpy PCR. We sequenced the ITS region for the *P. insidiosum* isolates obtained in the July collection to confirm the results of the ITSpy PCR. We obtained sequence from 14 isolates and BLAST analysis in GenBank indicated that seven isolates were identical to *P. insidiosum* Cluster I sequence types and seven were identical to the Cluster IV sequence type (22). A representative of each sequence type is available in GenBank as accession numbers MW517982 and MW517983. Cluster I sequences are typically recovered from clinical infections in humans and domestic animals in the Americas, whereas Cluster IV sequences have been recovered from the environment but have rarely been associated with disease (17). Both sequence types were found in the northern paddock and outside the grazing areas. None of the sequenced isolates were obtained from the southern paddock. Because we did not retain multiple isolates per site, we cannot determine the relative distribution of sequence types in the refuge.

In total, 30 spatially unique sites were determined positive for *P. insidiosum*. Most presences were at low salinity pools in salt marsh and brackish impoundments, and ephemeral ponds and water holes. Some sites were positive in June and July, while others that were positive in July were not detected as positive in June, and vice versa. Collection of two or four bottles of water per site in July likely increased our ability to detect *P. insidiosum* relative to the collection of a single sample of water in June. All but one of the sites where *P. insidiosum* was detected in June but not July had salinity above 10 parts per thousand (ppt) in July, and we did not detect *P. insidiosum* from any water with salinity >7 ppt. In June and July, *P. insidiosum* was detected at water temperatures ranging from 25 to 38°C. In September, *P. insidiosum* was detected in water of 21 and 22°C.

### Spatial Patterns of *P. insidiosum*

**Figure 3:**
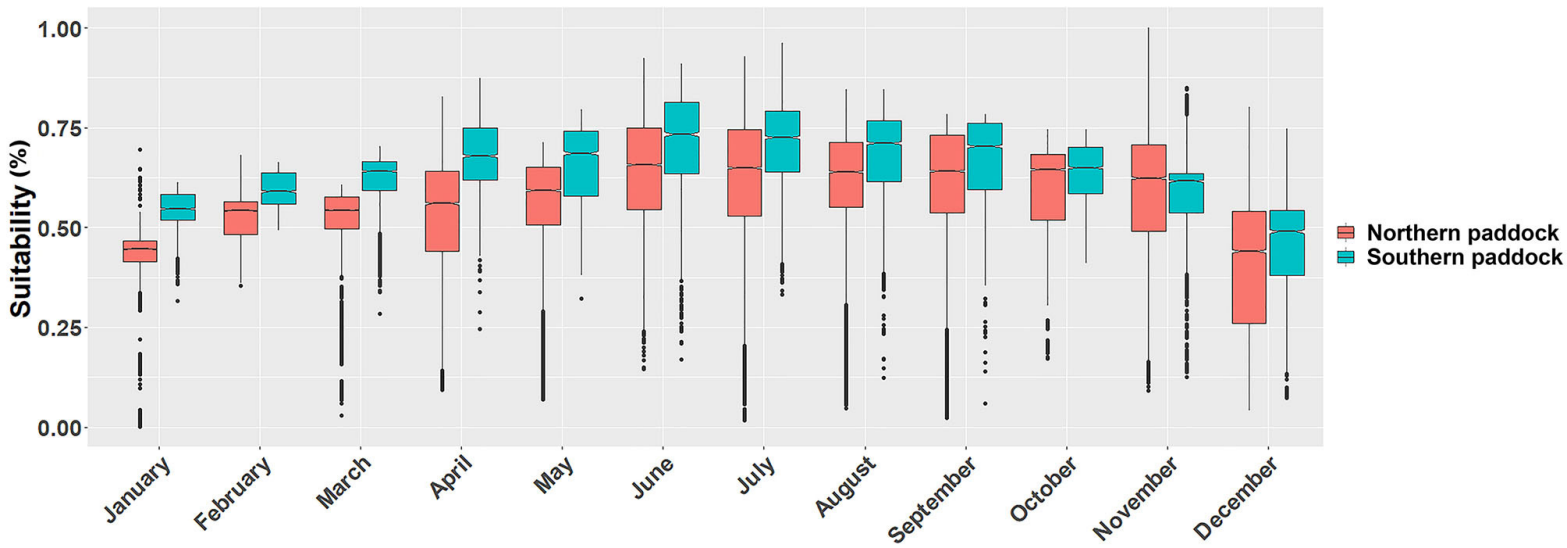
Monthly *P. insidiosum* suitability distributions. Each box-and-whisker plot represents the monthly suitability values for each grazing paddock. Y-axis represents the weighted distribution where the suitability distribution was simplified by dividing it by the number of cells (30 m resolution) of each paddock.

From the 136 samples collected during the period from June until September 2019, the 30 positive *P. insidiosum* samples (Figures 2A, 3A) were identified in a landscape characterized by freshwater wetlands mostly inhabited by submergent and emergent vegetation with limited salt tolerance. Likewise, tidal flooding influences the distribution of salt marsh plants that can be mostly found on the west side of the islands (Figure 2B).

### Ecological Niche Model (ENM) Overall Predictions

Model evaluation results showed that overall, the model that exhibited the best performance (ΔAICc = 0) was quadratic + threshold + hinge with a regularization multiplier of 1.9 (Supplementary Table 2). The overall prediction resulted from the dynamic model showed high proportion of suitable areas for *P. insidiosum* across the whole island (Figure 2B). When comparing the two grazing areas, we observed that the northern paddock showed the lowest proportion of suitable areas (Figures 2C,D). While the southern grazing paddock, which is mostly utilized for horse viewing, hosts a potentially much higher population with higher suitability areas, these areas consist mostly of tidal salt marsh and several ephemeral ponds and watering holes (Figure 2). The previously observed pattern was also supported by the annual ENM predictions (Supplementary Figure 2).

### Ecological Niche Model (ENM) Monthly Predictions

Ecological niche models showed substantial monthly variation in the predicted suitable areas across the island. We observed that the monthly fluctuation in the proportion of suitability affected both paddocks similarly, while the southern paddock produced a higher proportion of suitable areas, except during November (Figure 3).

### Environmental Drivers of *P. insidiosum* Distribution

ENM results showed that overall, the most influential predictors of *P. insidiosum* distribution were land-surface water, assessed through NDWI (median = 39.4%), minimum temperature (median = 26.8%), followed by mean annual temperature (median = 19.3%) (Table 1). However, we also observed that the level of importance of those three variables varied from month to month, for example, the importance of maximum temperature was observed to be lower during the warmer months (May–July) (Table 1).

**Table 1:** Variable importance for the annual and monthly ecological niche models.

**Month**	**Mean annual temperature**	**Minimum temperature**	**Maximum temperature**	NDWI^*^	**Cation exchange capacity**
January	3.2	34.4	13	45.3	4.1
February	14.3	21.9	15.9	44.7	3.2
March	19.8	28.1	9.9	41.2	1
April	7.3	31.3	16.8	41.7	2.9
May	25.4	28.4	5.9	38.5	1.8
June	19.3	37.1	2.6	39	2
July	24.2	26.8	5.6	40.6	2.8
August	17.2	23.5	10.4	45.8	3.1
September	21.5	23.1	13.2	38.9	3.3
October	19.3	29.1	8.3	39.2	4.1
November	22.1	23.1	12	37.7	5.1
December	16.8	26.6	12.4	39.4	4.8
Annual	19.5	23.5	13.4	38.5	5.1

* *Normalized Difference Water Index (NDWI)*.

*Warmer colors represent higher levels of importance (%)*.

To visualize the variation in the probability of presence (suitability) as each environmental variable is varied, we looked at the MaxEnt response curves of the annual model (Supplementary Figure 3). Our results showed that surface water showed a positive relationship with areas with higher suitability since the majority of hotspots are related to areas that are surrounding superficial water bodies. Minimum temperature showed a direct relationship with the observed suitability values. Likewise, mean annual temperature evidenced the highest peak of suitability at 22°C. The remaining variables showed lower levels of contribution to the ENM. For example, in relation to maximum temperature, we observed that suitability remained constant as temperature increases until reaching 30°C, from where it started to decline as the temperature increased. Finally, soil cation exchange capacity showed a peak at ~350 mmol(C)/kg.

### *P. insidiosum* Suitability Between Northern and Southern Paddocks

We compared the distribution of suitable sites between the northern and southern grazing paddocks. We observed statistically significant differences between the two grazing areas in every month (t_mean_=−37, *p* < 0.01). Our results also showed that the lowest suitability values were observed during the cold months, between December and March, while the highest levels during June, July, and August (Figure 3).

### Landscape Conditions Associated With the Highest Suitability Areas

Areas that showed higher suitability in our model were mostly associated with submergent and emergent herbaceous wetlands (33.7%), followed by woody wetlands (30.7%), and barren land (21.1%). These types of land cover are widely distributed across the island, with 38.1, 24.6, and 26.5% of coverage in the study area, respectively, being mostly distributed along the western side (Supplementary Table 4).

### Management Recommendations and Surveillance Sites

Our results showed that the majority of the refuge exhibits moderate to high suitability, although we observed a cold-spot between the two grazing areas. Proportionally, the southern paddock was more suitable for *P. insidiosum* than the northern paddock, and the summer months more suitable than the winter months. These observations could help guide exposure management and pathogen surveillance.

## Discussion

This study reconstructed the potential distribution of *P. insidiosum* in the Chincoteague National Wildlife Refuge, Assateague Island Unit located in the state of Virginia, United States. The distribution of positive water samples indicated a widespread prevalence of *P. insidiosum* swimming zoospores in the Refuge. Our ecological niche model predicted suitable areas for *P. insidiosum* zoosporulation across the Refuge, beyond the areas where the disease has been detected within the horse population. Novel areas predicted at risk include ephemeral wetlands associated with maritime forest and adjacent emergent freshwater/brackish marsh. The most dominant plant species associated with these habitats is *Spartina patens*, however, emergent vegetation within freshwater impoundments include, *Eleocharis sp*., *Bidens sp*., *Polygonum sp*., *Scirpus sp*., *Bacopa monnieri*, and *Phragmites australis*. We observed a higher potential exposure of the horse population to suitable conditions for the presence of *P. insidiosum* during June, July, and August, especially in the southern paddock.

Despite the global distribution exhibited by *P. insidiosum* (55, 56), a limited number of studies have examined its environmental preference, or external variables that promote its growth, survival and zoospore production (10, 22, 26, 57). Previous studies examined how *Pythium* spp. sporulation is triggered by specific environmental and host cues such as humidity and the presence of vegetation (58). Although zoospores require fresh water to disperse, it is important to consider that even a sheen of dew on a leaf surface or moist soil is sufficient for sporulation (22, 29). Our results showed that even though the majority of predicted areas appeared to be near a known water source, we found some risk areas in places with no permanent water bodies. This can be explained by the fact that *P. insidiosum*, like other *Pythium* species, can form drought-resistant oospores that germinate when an area is re-flooded (4). In relation to the host cues, it is well-known that *P. insidiosum* motile zoospores are chemotactically attracted to both plant and animal tissues (2, 4). Our results are consistent with plant material as a source of nutrition for *P. insidiosum* since we observed that suitable conditions were associated with non-emergent and emergent herbaceous wetlands, as well as by woody wetlands. We also found that these conditions are widely present in the ecoregion of middle Atlantic coastal forests where the Chincoteague National Wildlife Refuge is located (54, 59).

While *P. insidiosum* and some other *Pythium* species are known to exhibit peak growth rates at high temperatures (~37°C) (15, 30), our ENM monthly response curves suggested that low temperature can also play an important role as a limiting factor for explaining *P. insidiosum* distribution, and consequently, reducing the exposure of host populations during colder months (Supplementary Figure 3). Our results also evidenced significant differences among months in the predicted suitable areas, linked to annual seasonal variation in temperatures and water availability. The lowest suitability levels were associated with colder seasons (from December to March) and the highest suitability during June, July, and August when water levels in the freshwater impoundments are at their lowest for the benefit of migrating shorebirds. The summer months may be active months for *P. insidiosum* due to warm temperatures, but the water is drawn down in the impoundments during these months, leading to less surface water to support *P. insidiosum* populations.

In this study, we applied an ecological niche model (ENM) approach using the occurrences of *P. insidiosum* zoospores and high-resolution satellite imagery data. ENMs have been widely applied in epidemiology to predict species' potential distributions aiming to anticipate the future emergence of pathogens (60–66). This has been achieved by the characterization of the environmental requirements of the target species making use of satellite data, which has demonstrated to offer relevant guidance for local animal and human public health decision-making (67–69). Nevertheless, the main limitation of this study is that it relies on environmental variables that were processed as a monthly average, therefore, the predicted suitable areas should not be considered as physiological limits of tolerance of *P. insidiosum*.

Finally, this study highlights the imperative need to improve our ability to adequately define risk areas for pythiosis, given its severe and often fatal impact on infected animals despite its overall low incidence. Thus, the information generated here is a valuable first step toward informing pythiosis prevention and control plans in the Chincoteague National Wildlife Refuge and a first step toward defining high risk environments elsewhere.

## Conclusion

In this study, we examined the ecological conditions favoring *P. insidiosum*, by identifying its geographic and environmental distribution in the Chincoteague National Wildlife Refuge Island. Using an ecological niche model approach, we produced an annual high-resolution map to visualize the most suitable areas for the presence of infectious *P. insidiosum* zoospores. The suitability map could be used to identify areas of higher risk for surveillance and of lower risk for grazing. We also found that June, July, and August were the months that showed the highest suitability for *P. insidiosum* in both grazing areas, especially in the southern paddock, with potential elevated suitability in isolated wet areas throughout the summer months. The information generated in this study could be useful to design dynamic management plans aimed at reducing the risk of exposure to *P. insidiosum*.

## Data Availability Statement

The datasets presented in this article are not readily available. Requests to access the datasets should be directed to gmachad@ncsu.edu.

## Author Contributions

EG and GM conceived the study, designed the study, and secured the funding. EG, GM, and KH conducted field sampling. XW, KH, and EG processed the samples and did the laboratory isolations. MJ conducted data processing, cleaning, and data analysis. MJ, XW, EG, KH, and GM wrote and edited the manuscript. All authors discussed the results and critically reviewed the manuscript.

## Conflict of Interest

The authors declare that the research was conducted in the absence of any commercial or financial relationships that could be construed as a potential conflict of interest.
