# Corrigendum: The Potential Distribution of *Pythium insidiosum* in the Chincoteague National Wildlife Refuge, Virginia

**DOI:** 10.3389/fvets.2022.893668

**Published:** 2022-04-05

**Authors:** Manuel Jara, Kevin Holcomb, Xuechun Wang, Erica M. Goss, Gustavo Machado

**Affiliations:** ^1^Department of Population Health and Pathobiology, College of Veterinary Medicine, North Carolina State University, Raleigh, NC, United States; ^2^U.S. Fish & Wildlife Service, Chincoteague National Wildlife Refuge, Chincoteague, VA, United States; ^3^Plant Pathology Department and Emerging Pathogens Institute, University of Florida, Gainesville, FL, United States

**Keywords:** disease mapping, spillover, equine pythiosis, spatial epidemiology, ecological niche model

In the published article, there was an error in affiliation 3. Instead of “Mount Vernon Northwestern Washington Research & Extension Center Mount Vernon, Mount Vernon, WA, United States,” it should be “Plant Pathology Department and Emerging Pathogens Institute, University of Florida, Gainesville, FL, United States.”

The authors apologize for this error and state that this does not change the scientific conclusions of the article in any way. The original article has been updated.

## Publisher's Note

All claims expressed in this article are solely those of the authors and do not necessarily represent those of their affiliated organizations, or those of the publisher, the editors and the reviewers. Any product that may be evaluated in this article, or claim that may be made by its manufacturer, is not guaranteed or endorsed by the publisher.

