# Supplementary material for: The Potential Distribution of Pythium insidiosum in the Chincoteague National Wildlife Refuge, Virginia
Source: Front Vet Sci. 2021 Feb 19;8:640339. doi: 10.3389/fvets.2021.640339 (PMC7933582; doi:10.3389/fvets.2021.640339)
Supplement: Supplementary file 1 [file Data_Sheet_1.docx]

Supplementary Material

**Supplementary Table 1.** Variable selection and Variance Inflation Factor analysis (VIF) to assess spatial multicollinearity.

| **Variable** | **Unit** | **VIF** | **Source** |
| --- | --- | --- | --- |
| Mean annual temperature | °C | 5.98 | <https://clim-engine.appspot.com/climateEngine> |
| Minimum temperature | °C | 4.21 | <https://clim-engine.appspot.com/climateEngine> |
| Maximum temperature | °C | 5.95 | <https://clim-engine.appspot.com/climateEngine> |
| Normalized Difference Water Index (NDWI) | NDWI (green/SWRI2) | 3.56 | <https://clim-engine.appspot.com/climateEngine> |
| Soil cation exchange capacity | mmol(C)/kg | 2.12 | <https://soilgrids.org/> |

**Supplementary Table 2.** Model evaluation results based on the MaxEnt outputs that obtained the lowest ΔAICc. These models were tested under a wide variety of feature classes (linear= L, product= P, quadratic= Q, threshold= T, and hinge= H) and regularization multipliers.

| **Features** | **Regularization multiplier** | **AICc** | **ΔAICc** |
| --- | --- | --- | --- |
| QTH | 1.9 | 525.01 | 0.00 |
| Q | 2 | 525.06 | 0.05 |
| QT | 2 | 525.06 | 0.05 |
| QH | 2 | 525.06 | 0.05 |
| Q | 2 | 525.06 | 0.05 |
| Q | 0.7 | 525.95 | 0.95 |
| Q | 0.9 | 526.14 | 1.13 |
| Q | 1 | 526.25 | 1.24 |
| Q | 1.3 | 526.63 | 1.63 |
| QT | 1.3 | 526.63 | 1.63 |
| H | 1.5 | 526.74 | 1.73 |
| TH | 1.5 | 526.74 | 1.73 |
| QT | 1.9 | 476.53 | 1.73 |
| TH | 0.9 | 526.87 | 1.87 |
| Q | 1.5 | 526.95 | 1.94 |
| QT | 1.5 | 526.95 | 1.94 |
| QH | 1.9 | 476.81 | 2.01 |
| Q | 1.7 | 527.31 | 2.30 |
| QT | 1.7 | 527.31 | 2.30 |
| QTH | 1.9 | 477.23 | 2.43 |
| H | 1.7 | 527.52 | 2.51 |
| TH | 1.7 | 527.52 | 2.51 |
| Q | 0.1 | 528.28 | 3.27 |
| H | 1.9 | 528.38 | 3.37 |
| TH | 1.9 | 528.38 | 3.37 |
| Q | 0.3 | 528.57 | 3.56 |
| QP | 2 | 528.67 | 3.66 |
| QPT | 2 | 528.67 | 3.66 |
| PTQ | 2 | 528.67 | 3.66 |
| QPTH | 2 | 528.67 | 3.66 |
| QPH | 2 | 528.67 | 3.66 |
| Q | 0.5 | 528.72 | 3.71 |
| LP | 0.7 | 528.80 | 3.79 |
| H | 2 | 528.83 | 3.82 |
| TH | 2 | 528.83 | 3.82 |
| H | 1.3 | 528.84 | 3.83 |
| TH | 1.3 | 528.84 | 3.83 |
| L | 1.3 | 528.96 | 3.95 |
| LT | 1.3 | 528.96 | 3.95 |
| LP | 0.9 | 529.13 | 4.12 |
| L | 1.5 | 529.18 | 4.17 |
| LT | 1.5 | 529.18 | 4.17 |
| H | 0.9 | 529.23 | 4.22 |
| LP | 1 | 529.33 | 4.32 |
| PH | 1.5 | 529.34 | 4.33 |
| PTH | 1.5 | 529.34 | 4.33 |
| QP | 0.9 | 529.40 | 4.39 |
| LH | 1.5 | 529.42 | 4.41 |
| LTH | 1.5 | 529.42 | 4.41 |
| L | 1.7 | 529.44 | 4.43 |
| LT | 1.7 | 529.44 | 4.43 |
| QP | 1 | 529.55 | 4.54 |
| QH | 1.5 | 529.69 | 4.68 |
| QTH | 1.5 | 529.69 | 4.68 |
| LT | 1.9 | 529.72 | 4.71 |
| L | 1.9 | 529.72 | 4.71 |
| QTHL | 1.9 | 529.74 | 4.74 |
| LQ | 1.9 | 529.74 | 4.74 |
| LQT | 1.9 | 529.74 | 4.74 |
| LQH | 1.9 | 529.74 | 4.74 |
| LQ | 2 | 529.84 | 4.83 |
| LQT | 2 | 529.84 | 4.83 |
| LQH | 2 | 529.84 | 4.83 |
| QTHL | 2 | 529.84 | 4.83 |
| H | 1 | 529.86 | 4.85 |
| L | 2 | 529.88 | 4.87 |
| LT | 2 | 529.88 | 4.87 |
| LH | 1.7 | 529.96 | 4.95 |
| LTH | 1.7 | 529.96 | 4.95 |
| T | 1.9 | 530.02 | 5.01 |
| T | 2 | 530.02 | 5.01 |
| PTL | 1.3 | 530.05 | 5.04 |
| LP | 1.3 | 530.05 | 5.04 |
| QP | 1.3 | 530.06 | 5.06 |
| QPT | 1.3 | 530.06 | 5.06 |
| PTQ | 1.3 | 530.06 | 5.06 |
| PH | 1.7 | 530.12 | 5.11 |
| PTH | 1.7 | 530.12 | 5.11 |
| QH | 1.7 | 530.19 | 5.18 |
| QTH | 1.7 | 530.19 | 5.18 |
| QT | 1 | 530.30 | 5.29 |
| LPHT | 0.9 | 530.41 | 5.41 |
| QP | 1.5 | 530.46 | 5.45 |
| QPT | 1.5 | 530.46 | 5.45 |
| PTQ | 1.5 | 530.46 | 5.45 |
| PTH | 0.9 | 530.48 | 5.47 |
| LH | 1.9 | 530.54 | 5.54 |
| LTH | 1.9 | 530.54 | 5.54 |
| P | 0.3 | 530.59 | 5.59 |
| L | 0.1 | 530.61 | 5.60 |
| LP | 1.5 | 530.63 | 5.62 |
| PTL | 1.5 | 530.63 | 5.62 |
| L | 0.3 | 530.69 | 5.68 |
| P | 0.5 | 530.78 | 5.77 |
| LP | 0.3 | 530.82 | 5.82 |
| L | 0.5 | 530.84 | 5.83 |
| LTH | 2 | 530.85 | 5.84 |
| LH | 2 | 530.85 | 5.84 |
| QP | 1.7 | 530.90 | 5.89 |
| QPT | 1.7 | 530.90 | 5.89 |
| PTQ | 1.7 | 530.90 | 5.89 |
| PH | 1.9 | 530.97 | 5.97 |
| PTH | 1.9 | 530.97 | 5.97 |
| L | 0.7 | 531.06 | 6.05 |
| P | 0.7 | 531.07 | 6.06 |
| LTH | 0.9 | 531.16 | 6.15 |
| LP | 0.5 | 531.21 | 6.20 |
| PTL | 1.7 | 531.29 | 6.28 |
| LP | 1.7 | 531.29 | 6.28 |
| L | 0.9 | 531.35 | 6.34 |
| QPTH | 1.9 | 531.37 | 6.36 |
| QP | 1.9 | 531.37 | 6.36 |
| QPT | 1.9 | 531.37 | 6.36 |
| QPH | 1.9 | 531.37 | 6.36 |
| PTQ | 1.9 | 531.37 | 6.36 |
| PH | 2 | 531.43 | 6.42 |
| PTH | 2 | 531.43 | 6.42 |
| P | 0.9 | 531.46 | 6.45 |
| L | 1 | 531.52 | 6.51 |
| LQP | 2 | 531.57 | 6.57 |
| LQPT | 2 | 531.57 | 6.57 |
| LQPH | 2 | 531.57 | 6.57 |
| LPHQ | 2 | 531.57 | 6.57 |
| LQPTH | 2 | 531.57 | 6.57 |
| LPHT | 2 | 531.68 | 6.67 |
| P | 1 | 531.69 | 6.68 |
| PH | 1.3 | 531.75 | 6.74 |
| PTH | 1.3 | 531.75 | 6.74 |
| QH | 1.3 | 531.95 | 6.94 |
| QTH | 1.3 | 531.95 | 6.94 |
| PTL | 1.9 | 532.03 | 7.02 |
| LP | 1.9 | 532.03 | 7.02 |
| QT | 0.9 | 532.05 | 7.05 |
| T | 1.7 | 532.06 | 7.06 |
| TH | 1 | 532.24 | 7.24 |
| LQ | 0.1 | 532.26 | 7.25 |
| LP | 2 | 532.43 | 7.43 |
| PTL | 2 | 532.43 | 7.43 |
| P | 1.3 | 532.53 | 7.52 |
| LPHT | 1.5 | 532.58 | 7.57 |
| LQPT | 0.9 | 532.62 | 7.62 |
| LQP | 0.9 | 532.63 | 7.62 |
| PH | 0.9 | 532.71 | 7.70 |
| QPT | 0.9 | 532.75 | 7.74 |
| PTQ | 0.9 | 532.75 | 7.74 |
| LQP | 1 | 532.78 | 7.77 |
| LQT | 1.7 | 532.79 | 7.78 |
| LQ | 1.7 | 532.79 | 7.78 |
| LQH | 1.7 | 532.79 | 7.78 |
| QTHL | 1.7 | 532.79 | 7.78 |
| LT | 1 | 533.00 | 7.99 |
| P | 1.5 | 533.20 | 8.19 |
| PT | 1.5 | 533.20 | 8.19 |
| LQP | 1.3 | 533.29 | 8.28 |
| LQPT | 1.3 | 533.29 | 8.28 |
| PH | 1 | 533.35 | 8.34 |
| P | 0.1 | 533.40 | 8.39 |
| LH | 0.9 | 533.43 | 8.43 |
| LPHT | 1.7 | 533.44 | 8.43 |
| T | 1.5 | 533.57 | 8.56 |
| LQP | 1.5 | 533.69 | 8.68 |
| LQPT | 1.5 | 533.69 | 8.68 |
| LP | 0.1 | 533.83 | 8.82 |
| LH | 1 | 533.91 | 8.90 |
| P | 1.7 | 533.96 | 8.95 |
| PT | 1.7 | 533.96 | 8.95 |
| QPH | 1.7 | 534.08 | 9.07 |
| QPTH | 1.7 | 534.08 | 9.07 |
| LQP | 1.7 | 534.12 | 9.12 |
| LQPT | 1.7 | 534.12 | 9.12 |
| LQ | 0.3 | 534.15 | 9.15 |
| LQPT | 1 | 534.21 | 9.20 |
| QPT | 1 | 534.34 | 9.33 |
| PTQ | 1 | 534.34 | 9.33 |
| LQ | 0.5 | 534.43 | 9.42 |
| QTHL | 0.9 | 534.47 | 9.46 |
| LPHT | 1.9 | 534.50 | 9.49 |
| LQP | 1.9 | 534.60 | 9.59 |
| LQPT | 1.9 | 534.60 | 9.59 |
| LQPH | 1.9 | 534.60 | 9.59 |
| LPHQ | 1.9 | 534.60 | 9.59 |
| LQPTH | 1.9 | 534.60 | 9.59 |
| LQ | 0.7 | 534.65 | 9.64 |
| T | 1.3 | 534.68 | 9.67 |
| PT | 1.9 | 534.81 | 9.80 |
| P | 1.9 | 534.81 | 9.80 |
| LQ | 0.9 | 534.91 | 9.90 |
| LQP | 0.5 | 534.92 | 9.91 |
| LQ | 1 | 535.06 | 10.05 |
| LT | 0.9 | 535.11 | 10.10 |
| PT | 2 | 535.27 | 10.26 |
| P | 2 | 535.27 | 10.26 |
| LQP | 0.1 | 535.34 | 10.33 |
| LPHT | 1.3 | 535.38 | 10.37 |
| T | 1 | 535.40 | 10.39 |
| PT | 1.3 | 535.41 | 10.40 |
| LQ | 1.3 | 535.57 | 10.56 |
| LQT | 1.3 | 535.57 | 10.56 |
| LH | 1.3 | 535.57 | 10.56 |
| LTH | 1.3 | 535.57 | 10.56 |
| PTL | 0.9 | 535.66 | 10.65 |
| T | 0.1 | 485.62 | 10.82 |
| H | 0.1 | 485.65 | 10.85 |
| LT | 0.1 | 485.65 | 10.85 |
| LH | 0.1 | 485.65 | 10.85 |
| QT | 0.1 | 485.66 | 10.86 |
| LQH | 1.5 | 535.89 | 10.88 |
| QTHL | 1.5 | 535.89 | 10.88 |
| LQT | 1.5 | 535.97 | 10.96 |
| LQ | 1.5 | 535.97 | 10.96 |
| LPHT | 1 | 536.20 | 11.19 |
| QH | 0.1 | 486.00 | 11.20 |
| PT | 0.1 | 486.00 | 11.20 |
| PH | 0.1 | 486.00 | 11.20 |
| PTH | 1 | 536.24 | 11.23 |
| TH | 0.1 | 486.08 | 11.28 |
| LQH | 0.9 | 536.77 | 11.76 |
| LTH | 1 | 536.78 | 11.77 |
| T | 0.9 | 536.81 | 11.80 |
| LQT | 0.1 | 486.79 | 11.99 |
| LQH | 0.1 | 486.79 | 11.99 |
| QPT | 0.1 | 486.79 | 11.99 |
| QPH | 0.1 | 486.87 | 12.07 |
| QH | 0.9 | 537.26 | 12.25 |
| LQH | 1 | 537.29 | 12.29 |
| PTL | 0.1 | 487.17 | 12.37 |
| PTQ | 0.1 | 487.17 | 12.37 |
| LQT | 1 | 537.51 | 12.50 |
| PTL | 1 | 537.56 | 12.55 |
| PTH | 0.1 | 487.44 | 12.63 |
| LQPH | 1.7 | 537.69 | 12.68 |
| LPHQ | 1.7 | 537.69 | 12.68 |
| LQPTH | 1.7 | 537.69 | 12.68 |
| LTH | 0.1 | 487.53 | 12.73 |
| QTH | 0.1 | 487.53 | 12.73 |
| LQPT | 0.1 | 487.53 | 12.73 |
| LQPH | 0.1 | 487.56 | 12.76 |
| QH | 1 | 537.80 | 12.79 |
| QPTH | 0.1 | 487.63 | 12.83 |
| LPHQ | 0.1 | 487.63 | 12.83 |
| LQP | 0.3 | 538.07 | 13.06 |
| LPHT | 0.1 | 487.95 | 13.15 |
| QTHL | 0.1 | 487.95 | 13.15 |
| LQPTH | 0.1 | 487.95 | 13.15 |
| QT | 0.3 | 487.95 | 13.15 |
| PT | 0.3 | 488.32 | 13.52 |
| TH | 0.3 | 488.32 | 13.52 |
| LQT | 0.3 | 488.32 | 13.52 |
| QPT | 0.3 | 488.54 | 13.74 |
| PTQ | 0.3 | 488.71 | 13.91 |
| PTH | 0.3 | 488.71 | 13.91 |
| LTH | 0.3 | 488.73 | 13.93 |
| QTH | 0.3 | 488.73 | 13.93 |
| LQPT | 0.3 | 488.73 | 13.93 |
| PT | 1 | 538.94 | 13.94 |
| LQPTH | 0.9 | 538.98 | 13.97 |
| QPTH | 0.9 | 539.08 | 14.08 |
| T | 0.7 | 539.12 | 14.11 |
| QP | 0.5 | 539.12 | 14.11 |
| QPTH | 0.3 | 489.05 | 14.25 |
| LPHT | 0.3 | 489.05 | 14.25 |
| QTHL | 0.3 | 489.05 | 14.25 |
| LQPTH | 0.3 | 489.05 | 14.25 |
| QTH | 0.9 | 539.36 | 14.35 |
| H | 0.7 | 539.53 | 14.53 |
| PH | 0.7 | 539.63 | 14.62 |
| LQP | 0.7 | 539.65 | 14.64 |
| QP | 0.7 | 539.78 | 14.77 |
| QPTH | 1.3 | 540.04 | 15.03 |
| QPH | 1.3 | 540.04 | 15.03 |
| LPHT | 0.7 | 540.37 | 15.36 |
| PTH | 0.7 | 540.37 | 15.36 |
| LQH | 0.7 | 540.43 | 15.42 |
| QH | 0.7 | 540.51 | 15.50 |
| LQPTH | 0.7 | 540.53 | 15.52 |
| TH | 0.7 | 540.62 | 15.61 |
| QTHL | 1 | 540.73 | 15.72 |
| QPTH | 1.5 | 541.02 | 16.01 |
| QPH | 1.5 | 541.02 | 16.01 |
| QTH | 1 | 541.10 | 16.09 |
| LQPH | 0.9 | 541.20 | 16.19 |
| LPHQ | 0.9 | 541.20 | 16.19 |
| QPH | 0.9 | 541.27 | 16.26 |
| QTH | 0.7 | 541.63 | 16.62 |
| LQPH | 1 | 541.89 | 16.88 |
| LPHQ | 1 | 541.89 | 16.88 |
| QPH | 1 | 541.96 | 16.95 |
| QT | 0.7 | 542.81 | 17.80 |
| QP | 0.3 | 542.91 | 17.91 |
| LQH | 1.3 | 543.84 | 18.83 |
| QTHL | 1.3 | 543.84 | 18.83 |
| PT | 0.9 | 544.52 | 19.51 |
| LQPH | 1.3 | 544.64 | 19.63 |
| LPHQ | 1.3 | 544.64 | 19.63 |
| LQPTH | 1.3 | 544.64 | 19.63 |
| LH | 0.7 | 545.25 | 20.24 |
| LQPH | 1.5 | 545.62 | 20.62 |
| LPHQ | 1.5 | 545.62 | 20.62 |
| LQPTH | 1.5 | 545.62 | 20.62 |
| LQT | 0.9 | 545.66 | 20.65 |
| QP | 0.1 | 545.81 | 20.80 |
| LQPTH | 1 | 545.93 | 20.92 |
| QPTH | 1 | 546.00 | 20.99 |
| QPTH | 0.7 | 546.77 | 21.76 |
| QTHL | 0.7 | 546.88 | 21.87 |
| LQT | 0.7 | 547.79 | 22.78 |
| PT | 0.7 | 548.98 | 23.97 |
| LT | 0.7 | 550.95 | 25.94 |
| LQPH | 0.7 | 551.23 | 26.22 |
| LPHQ | 0.7 | 551.23 | 26.22 |
| QPH | 0.7 | 551.31 | 26.30 |
| LTH | 0.7 | 554.09 | 29.08 |
| H | 0.5 | 554.46 | 29.45 |
| LQPT | 0.7 | 555.32 | 30.31 |
| QH | 0.5 | 555.97 | 30.96 |
| QT | 0.5 | 556.29 | 31.28 |
| LQT | 0.5 | 565.32 | 40.31 |
| PTL | 0.7 | 565.38 | 40.37 |
| QPT | 0.7 | 573.90 | 48.89 |

**Supplementary Table 3.** Comparison between the predicted suitability areas for *Pythium insidiosum* between paddocks.

| **Variable** | **t** | **df** | **p-value** |
| --- | --- | --- | --- |
| January | 81.3 | 20924 | 0.000 |
| February | 64.8 | 20924 | 0.000 |
| March | 58.0 | 20924 | 0.000 |
| April | 56.2 | 20924 | 0.000 |
| May | 37.8 | 20924 | 0.000 |
| June | 27.8 | 20924 | 0.000 |
| July | 28.1 | 20924 | 0.000 |
| August | 26.4 | 20924 | 0.000 |
| September | 23.6 | 20924 | 0.000 |
| October | 19.6 | 20924 | 0.000 |
| November | 2.5 | 20924 | 0.012 |
| December | 17.3 | 20924 | 0.000 |

**Supplementary Table 4.** Comparison between land cover representation in the Chincoteague National Wildlife Refuge and in the areas classified as higher suitability for *Pythium insidiosum* presence (mean annual values).

| **Land cover type** | **% in island** | **% high suitability** |
| --- | --- | --- |
| Barren land (rock/sand/clay) | 26.5 | 21.1 |
| Cultivated crops | 0.1 | 0.0 |
| Deciduous forest | 0.5 | 0.2 |
| Developed, high intensity | 0.0 | 0.0 |
| Developed, low intensity | 0.5 | 0.8 |
| Developed, medium intensity | 0.1 | 0.1 |
| Developed, open space | 2.1 | 4.2 |
| Emergent herbaceous wetlands | 38.1 | 33.7 |
| Evergreen forest | 6.7 | 8.9 |
| Grasslands/herbaceous | 0.3 | 0.1 |
| Mixed forest | 0.2 | 0.1 |
| Shrubs | 0.3 | 0.1 |
| Woody wetlands | 24.6 | 30.7 |

**
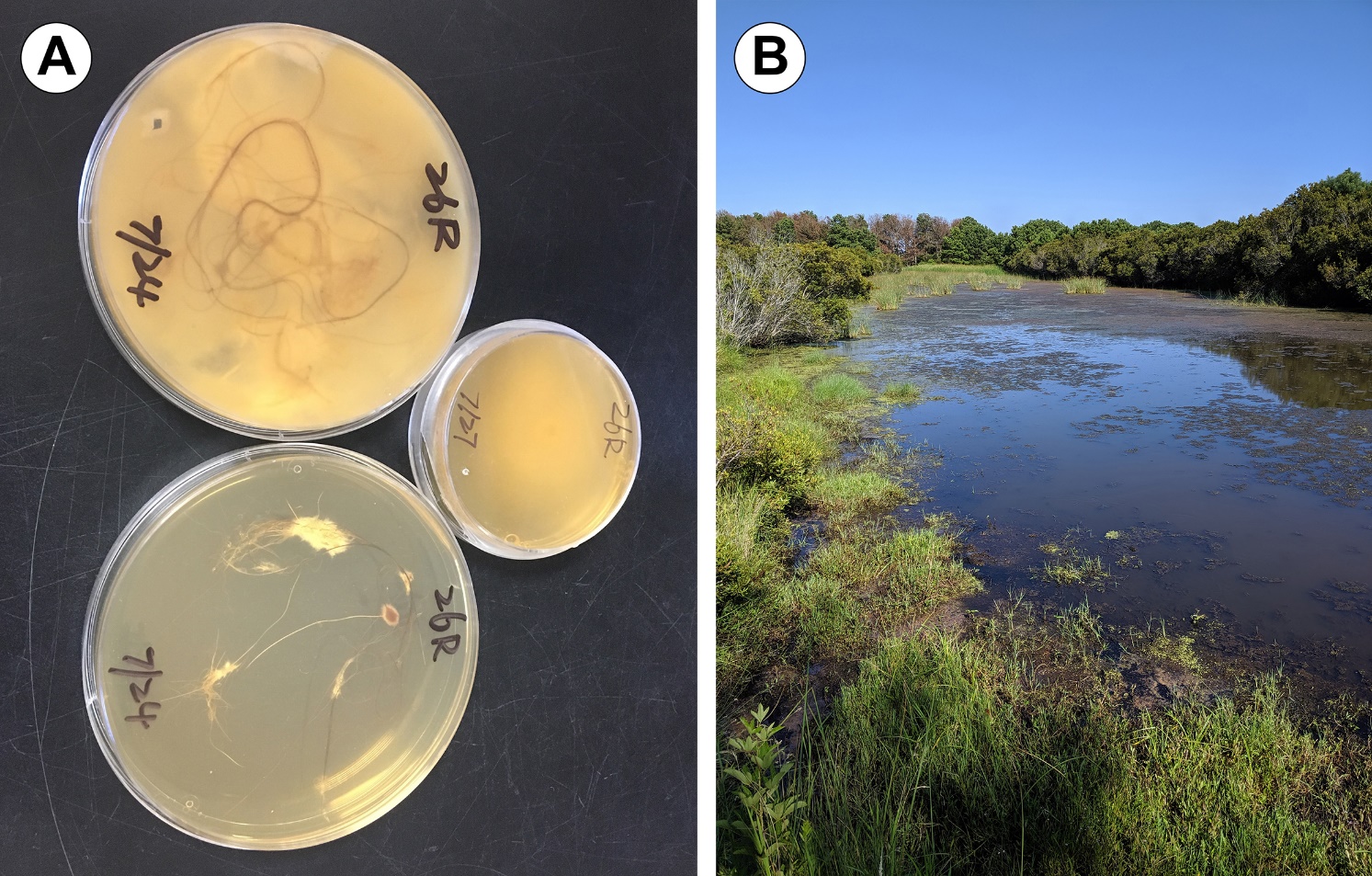
**

**Supplementary Figure 1.** A) *Pythium* growth on Sabouraud dextrose agar from one of two environmental samples taken at a site in July (large Petri dishes), from which *P. insidiosum* was subcultured (small Petri dish). B) Wetland area illustrating one of the positive sampling sites.

**
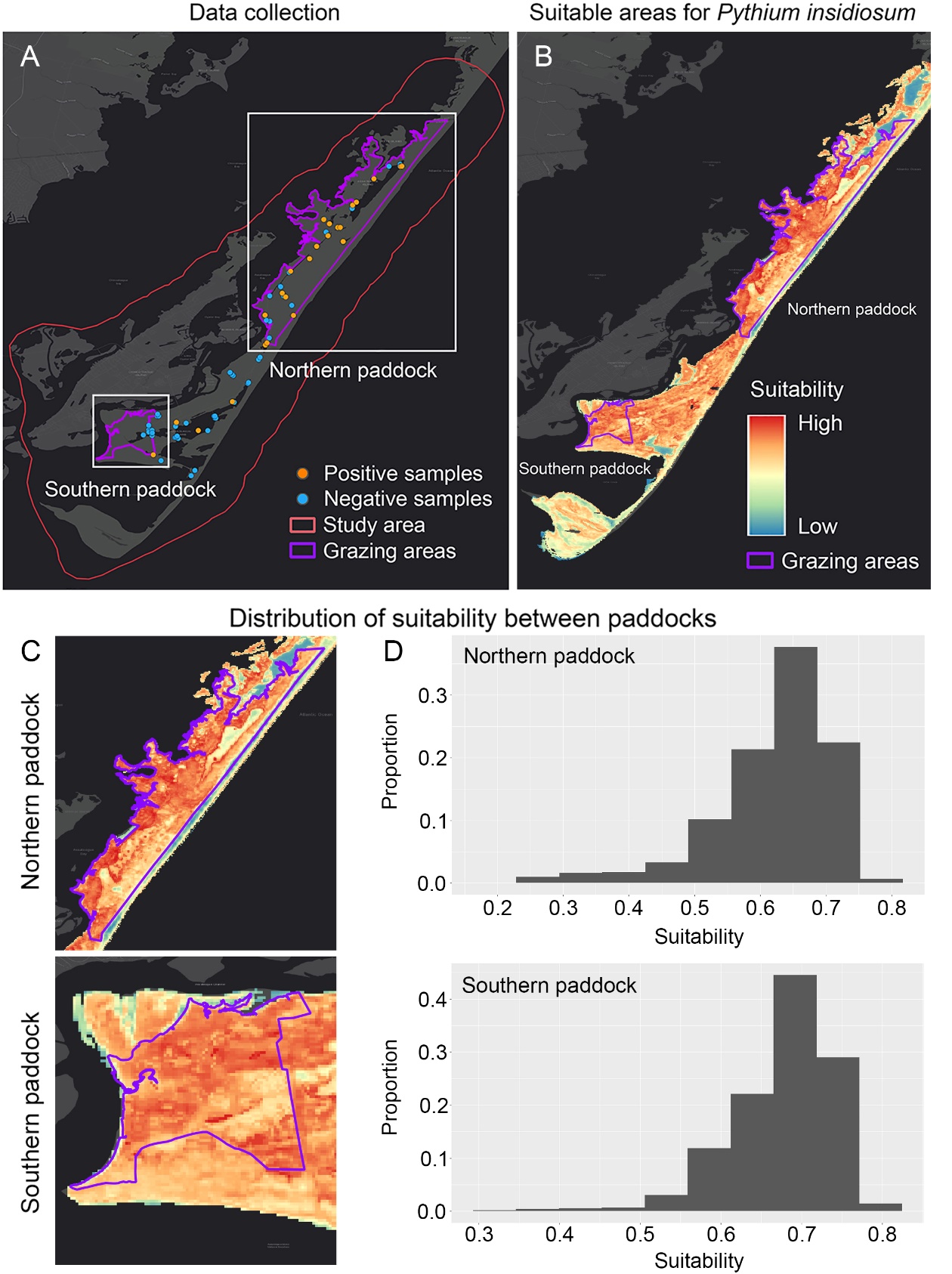
**

**Supplementary Figure 2.** Annual *Pythium insidiosum* suitable areas in Chincoteague National Wildlife Refuge. A) shows positive and negative *P. insidiosum* detections in both grazing paddocks, delimited by purple lines. B) shows the ecological niche model continuous results for the whole study area, and the suitability of each paddock (C). D) shows the comparison of the predicted suitability weighted distribution for *P. insidiosum* for each paddock, where suitability values range from 0 to 1, with 1 being the most suitable.

**
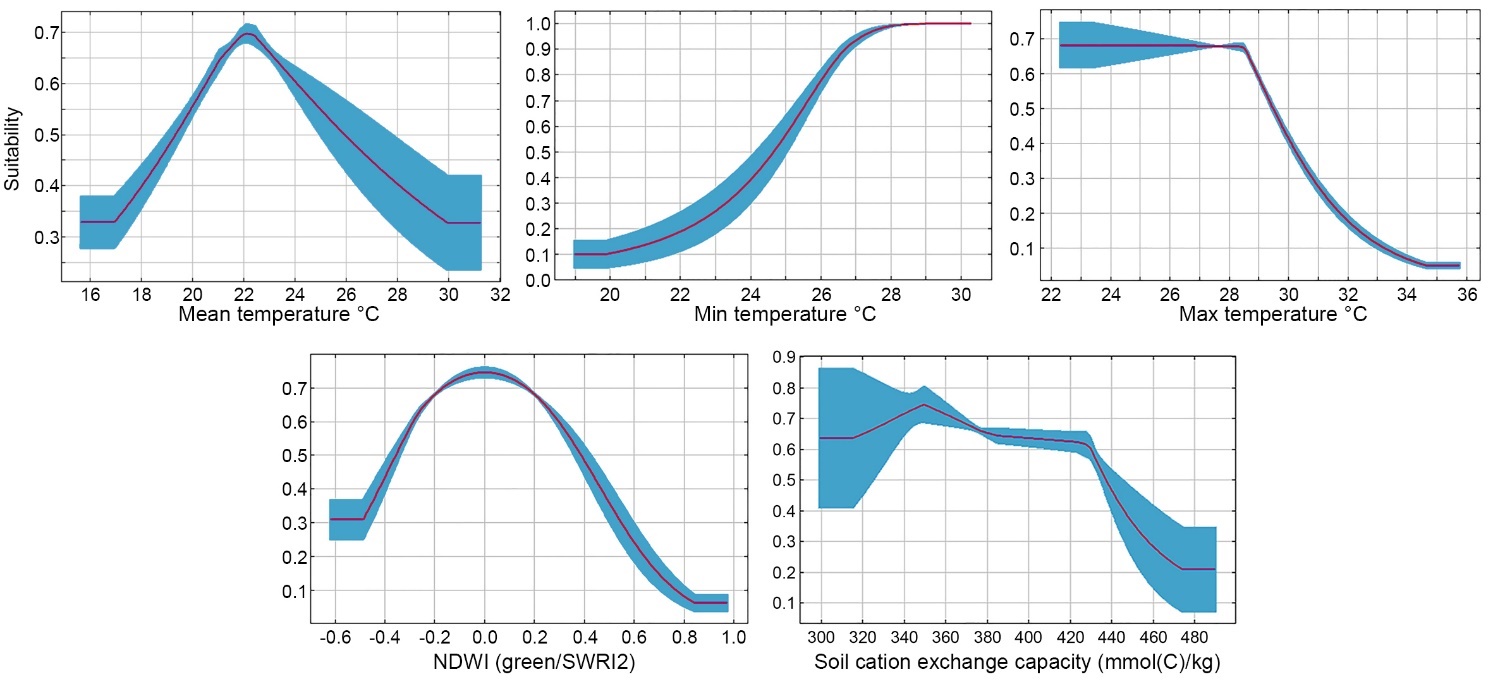
**

**Supplementary Figure 3.** Response curves of the different environmental variables used in the ENM (annual predictions). Response curves (red line) show the mean response of all the MaxEnt predictions (red) and the mean +/- one standard deviation (blue area).
